# Static Baropodometry for Assessing Short-Term Functional Outcome after Unilateral Total Knee Arthroplasty: Exploring Correlation between Static Plantar Pressure Measurements and Self-Reported Outcomes

**DOI:** 10.3390/jcm12216917

**Published:** 2023-11-03

**Authors:** Dimitrios Ntourantonis, Ioanna Lianou, Ilias Iliopoulos, Konstantinos Pantazis, Panagiotis Korovessis, Elias Panagiotopoulos

**Affiliations:** 1 Emergency Department, University Hospital of Patras, 26504 Patras, Greece; 2 Department of Medicine, School of Health Sciences, University of Patras, 26504 Patras, Greece; 3 Department of Orthopaedics, General Hospital of Patras, 26332 Patras, Greece; jolianou@hotmail.com (I.L.);; 4 Department of Orthopaedics, Aimis Healthcare Group, Larnaca 6309, Cyprus; iliopoulos.d.il@gmail.com; 5 Department of Orthopaedics, Central Hospital of Karlstad, 65230 Karlstad, Sweden; pantazis.kon@gmail.com; 6 Department of Orthopaedics, University Hospital of Patras, 26504 Patras, Greece; ecpanagi@upatras.gr

**Keywords:** static baropodometry, TKA, femorotibial angle, knee alignment, plantar pressure measurements, functional outcomes, KOOS, short term

## Abstract

This study aimed to investigate the association between objective baropodometric and radiological measurements and patient self-reported functional outcomes, assessed through the Knee Injury and Osteoarthritis Outcome Score (KOOS). Additionally, it sought to evaluate the effectiveness of static baropodometry in predicting short-term KOOS results following unilateral total knee arthroplasty (TKA). We conducted a prospective single-center study involving 32 patients who underwent unilateral TKA for knee osteoarthritis (KOA). Patients were evaluated both preoperatively and six months postoperatively, utilizing objective measurements derived from static baropodometric analysis in a normal, relaxed, bipedal standing position using a multi-platform Plantar Pressure Analysis System (PPAS) and radiographic measurements of the femorotibial angle (FTA) and subjective assessments through the national validated version of the KOOS. The study found an insignificant average correction of −0.69° ± 4.12° in the preoperative FTA at the sixth month after TKA. Moreover, there were no significant differences in the KOOS based on different types of knee alignment (KA) both pre- and postoperatively (*p* > 0.05). No significant correlations were observed between the KOOS, and total average affected and unaffected plantar pressures (TAAPP and TAUPP) pre- and postoperatively, as well as KA pre- and postoperatively. However, significant changes were observed in TAAPP and TAUPP measurements after unilateral TKA. TAAPP demonstrated a significant increase postoperatively (mean change (SD) = 18.60 (47.71); *p* = 0.035). In conclusion, this study found no significant correlation between KA, static baropodometric measurements, including pre- and postoperative differences, and KOOS outcomes. Therefore, static plantar pressure measurements alone might not serve as a reliable predictor of short-term clinical outcomes after unilateral TKA, as reported by patients.

## 1. Introduction

Total knee arthroplasty (TKA) is one of the commonest, most successful, and cost-effective treatment options performed worldwide for function improvement and pain relief mainly in patients with knee osteoarthritis (KOA) of end-stage. This treatment option was first used in the 1970s [1,2,3]. Nowadays, more than 700,000 patients undergo TKA annually in the United States, [4] and this amount is expected to increase by around 673% by 2030 [5]. Similarly, more than 100,000 TKAs are performed in the United Kingdom every year [6]. The increase in the incidence of primary osteoarthritis causes an increase in the number of these replacements, while the number of inflammatory arthropathies tended to be unchanged over the past ten years due to the usage of efficient antirheumatic medication [7].

The complexity of knee anatomy and the limited knowledge on it, especially during the first years of TKA introduction, made it difficult to restore kinematics and knee anatomy, while the main goal was to expand the survivorship of the implant [8]. Consequently, the initial procedure included rectangular flexion and extension gaps, extensive soft tissue release, a neutral mechanical axis, and a joint line perpendicular to the mechanical axis [9], with Insall being a pioneer in performing TKA with mechanical alignment [10]. Until recent years, it was believed that the restoration of the axis as close to neutral after the procedure was the key to improved clinical results and implant survival. Misalignment has been correlated with diminished implant survival rates, elevated wear rates, suboptimal functional outcomes, and premature failure, especially in the context of earlier polyethylene and implant designs [11,12,13,14,15,16,17]. On the other hand, recent reports have demonstrated comparable functional outcomes and implant survival rates between patients with constitutional and mechanically neutral alignments [18,19,20].

The evaluation of the improvement in physical performance and patients’ satisfaction following TKA is achieved by a large number of outcome measures (self-reported functional outcome scores), including the Knee Injury and Osteoarthritis Outcome Score (KOOS), Western Ontario and McMaster Universities Osteoarthritis (WOMAC), and Oxford Knee Score (OKS) [21]. Even though TKA is a “popular” orthopedic procedure, patients’ satisfaction is conflicting, and up to 20% of them are dissatisfied [22]. This could be explained by great differences in kinematics, resulting from deficiencies in the knowledge of anatomy and biomechanics [23]. Moreover, satisfaction rate is related to several different factors including younger or older age, female gender, worse preoperative pain scores, the presence of rheumatoid arthropathy, and patients’ personality (pessimism) [1,7,24,25,26].

Patients with KOA requiring TKA commonly present with abnormalities to the ankle and to the foot alignment. It appears plausible that certain adaptive alterations take place in both joints as a response to abnormalities in the other [27,28,29]. When there is a deviation in alignment toward varus or valgus in the knee, adjustments might occur in the hindfoot to bring back a balanced alignment in the coronal plane of the hip, knee, and ankle [27,29]. However, the precise mechanisms through which the foot and ankle adapt to a specific varus or valgus deformity of the knee are not yet entirely comprehended [28,30]. Chandler and Moscal have reported that TKA leads to alterations in hindfoot alignment in a consistent and predictable manner [31].

As the human foot is the basis of support, plantar pressure measurements can potentially assess dysfunctions not only related to the foot but to the knee joint as well, and it might be valuable in terms of postural assessment. Baropodometry is a straightforward analytical technique that utilizes pressure sensors embedded in the ground to assess the distribution of foot pressure during standing or on the course of a typical gait cycle. What necessitates the use of plantar pressure measurements is the application of Newton’s third law, where action and reaction are equivalent. As individuals stand or walk, reciprocal forces are transmitted between the body and the ground. Evaluating these forces at ground contact can aid in understanding the external burdens placed on the human body during typical activities like standing or walking, as well as during more intense scenarios such as sports [32].

As we have already mentioned, the correction of knee joint malalignment after TKA initially affects the alignment of the foot [27,28,29,30,31] and, as a result of these adaptations, the data collected from Plantar Pressure Analysis Systems (PPASs) [29,33,34,35]. Studies have reported that TKA changes patients’ standing patterns compared to the preoperative results, and the difference is noted soon after operation [34,35,36,37]. Although this simple evaluation can improve understanding of joint biomechanics following TKA and aid rehabilitation planning [34], only a few studies have focused on the use of baropodometry to estimate the impact of TKA on postural adaptation and coronal alignment [34,35,38,39,40]. In contrast, baropodometry has been widely used in studies of several pathologies including diabetic foot and neurological disorders [41].

Given the known discrepancy between patient satisfaction and clinical outcomes as rated by clinicians [42] and evaluated by measurement devices and functional scales [43,44], baropodometry could provide an objective and quantitative assessment of the impact of TKA on postural adaptation [34] and potentially on self-reported functional outcome scores if these parameters are directly related to the restoration of the axis after unilateral TKA.

The primary objective of the present study was twofold: Firstly, we aimed to explore the relationship between self-reported functional outcome scores as collected through the KOOS and disparities in knee alignment (KA), specifically documented through alterations in the femorotibial angle (FTA) following unilateral TKA. Secondly, the study aimed to investigate any potential connection between static baropodometric analysis, KA, and KOOS outcomes. Considering that recent studies have demonstrated similar results between mechanical and constitutional alignment, our hypothesis was that static baropodometric results would not exhibit a direct correlation with KOOS outcomes.

## 2. Materials and Methods

### 2.1. Study Design

This prospective, single-center study included patients diagnosed with primary KOA scheduled for unilateral TKA with a posterior cruciate ligament retaining (CR) implant (SIGMA™ CR 150, Depuy Synthes, Warsaw, IN, USA). The study protocol was approved by the Institutional Review Board (IRB numbers: 187/21114; 265/7623). All patients provided written informed consent to participate. The pre-hoc power analysis was performed using the G-power 3.1 statistical software [45]. To estimate the required sample size, Cohen’s effect size dz (0.5), α (0.05), and 1-β (0.8) error probability values were used. The minimum required sample size was calculated as 27.

### 2.2. Participants

Inclusion criteria: (a) primary KOA grades III–IV according to Kellgren–Lawrence Classification (KLC) [46]; (b) ability to walk independently prior to surgery; (c) age > 60 years. Exclusion criteria: (a) existing neurological deficits that could influence the gait or standing patterns; (b) prior orthopedic surgery on the lower limb or/and spine; (c) lack of co-operation; (d) major intra- and postoperative surgical complications, including (i) implantation of any other type of prosthesis, (ii) postoperative infections, and (iii) periprosthetic fractures; (e) any other condition during the inclusion to the protocol period with an impact on gait or standing patterns, such as trauma to the lower limbs and/or spine or neurological conditions. Each participant underwent preoperative assessment, which included standing knee X-rays, static baropodometric measurements, and KOOS evaluation. This assessment was repeated on the sixth postoperative month.

**Scheme 1:**
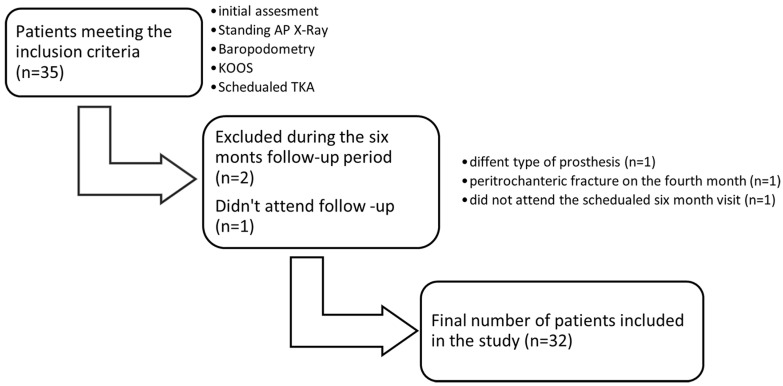
Study flowchart.

A total of 35 patients who met the inclusion criteria were initially enrolled. During the six-month follow-up period, one patient was excluded immediately after TKA due to the use of a posterior stabilized prosthesis, one sustained an ipsilateral peritrochanteric hip fracture in the fourth postoperative month, and one patient did not attend the six-month reevaluation visit (Scheme 1). The final sample consisted of 32 patients (68.8% females, *n* = 22) with mean age of 72.8 years (SD = 6.6 years). Mean body mass index (BMI) was 27.1 kg/m^2^ (SD = 2.0 kg/m^2^), and 81.3% of participants were overweight. Seventeen out of thirty-two patients had an affected left knee while the majority of patients (*n* = 23) were classified as grade 4 according to KLC [34]. Further demographics of our study group are presented in Table 1. The group of excluded patients (*n* = 3) had similar characteristics in terms of age, BMI, percentage of males (M) and females (F), KOOS, and TAAPP–TAUPP when compared to the analyzed group.

### 2.3. Surgical Technique

All TKAs were performed at the authors’ institute by a single experienced surgeon. A standard midline skin incision and medial parapatellar approach were used. Tibial and femoral components were fixed with cement, and the patella was resurfaced routinely. The patients’ rehabilitation program was standardized and followed our institute’s principles.

### 2.4. KOA and FTA Assessment

**Figure 1:**
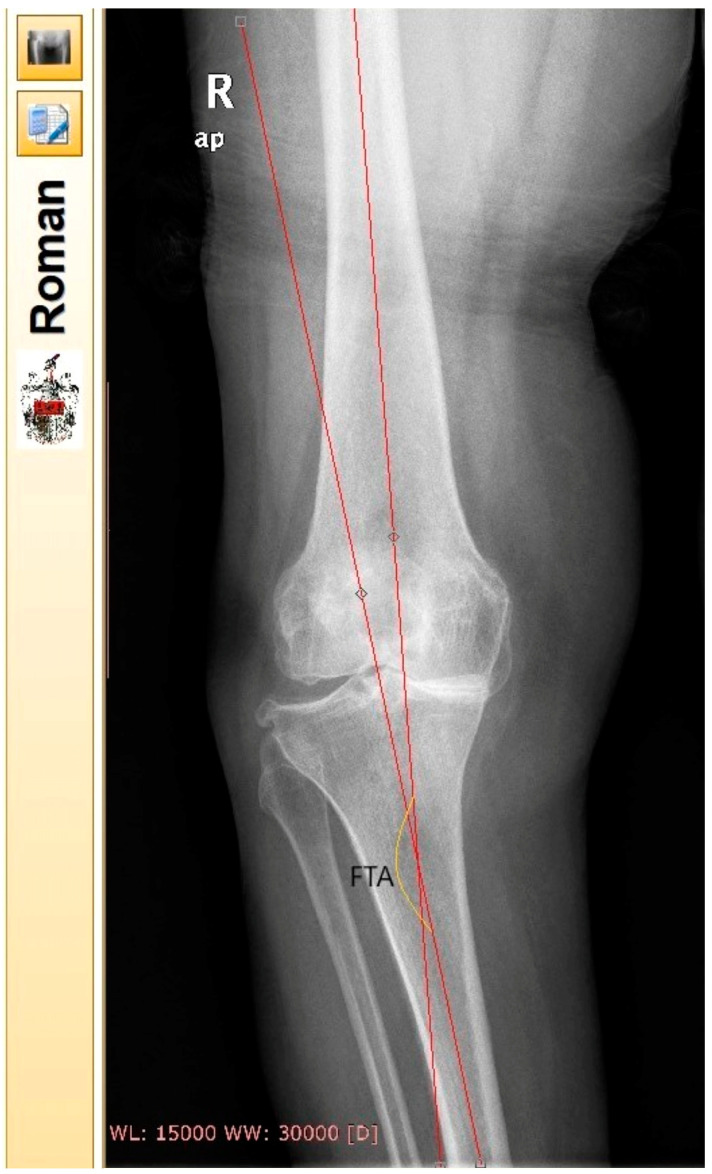
FTA measurement in the environment of Roentgen Monographic Analysis (RoMAn) free-to-share software version V1.70. The femoral anatomic axis was drawn by locating the midway between the femoral cortex in two points. The first one as far proximally as the radiograph allows and the second one 10 cm above the joint line. The same procedure was followed for the tibial axis. In this specific X-ray, FTA was calculated in a varus knee.

Preoperative KOA classification was performed by the surgeon who listed the patients for surgery. All patients underwent anteroposterior (ap) standing digital radiographs of the knee joints pre- and six months postoperatively (large film, 14 × 17 inch). FTA of the affected knee joint (AKJ) was measured according to Petersen and Engh [47]. Two independent blinded observers who did not participate in any other part of the study performed the FTA measurements. The sequence of X-rays and the file names were randomly generated. Same measurements were repeated by the observers with an interval of 15 days for both radiological assessments (pre- and six months postoperatively). The reported FTA pre- and postoperatively was calculated as the average of observers’ measurements. For the measurement of FTA, Roentgen Monographic Analysis (RoMAn) [48,49] free-to-share software version V1.70 was used (Robert Jones and Agnes Hunt Orthopaedic Hospital) (Figure 1). Each knee was categorized pre- and postoperatively as varus (VR), valgus (VL), or neutral (N) [50,51]. N knees were characterized as the ones with an FTA between 172.8° and 177.6°, VR as the ones with FTA > 177.6°, and VL knees as the ones with FTA < 172.8.

### 2.5. Baropodometric Analysis

**Figure 2:**
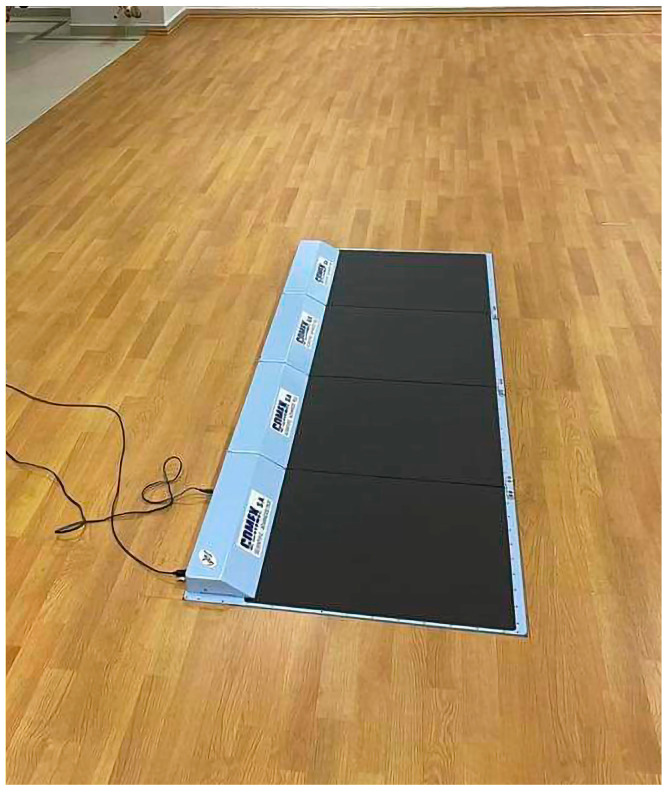
The Multiplatform System by Comex (rebranded Loran Engineering Srl MPS) that was used for static baropodometric measurements.

**Figure 3:**
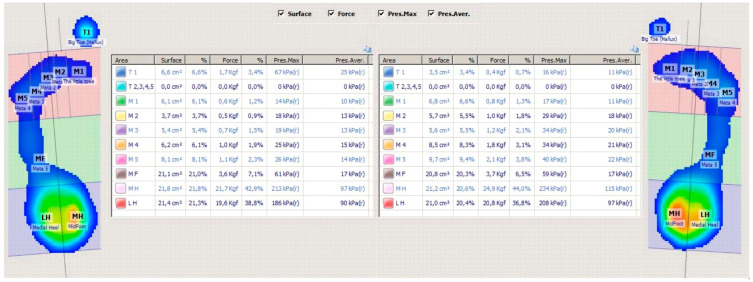
Data on baropodometry using “Biomech Studio” Version 1 (Loran Engineering Srl) [53] software were exported, providing measurements of pressures in kilopascals (kPa) in ten specific areas of the feet as follows: medial hindfoot (MH), lateral hindfoot (LH), big toe (T1), second to fifth toes (T2–T5), first metatarsal head (M1), second metatarsal head (M2), third metatarsal head (M3), fourth metatarsal head (M4), fifth metatarsal head (M5), midfoot (MF), medial hindfoot (MH), and lateral hindfoot (LH).

Data from barefoot plantar pressure (PP) distribution analysis in a static standing position were also collected at the same intervals. The measurements were conducted using a multi-platform PPAS (Multiplatform System (MPS) by Comex (rebranded Loran Engineering Srl MPS)) [52,53] (Figure 2). The system comprises four separate platforms that can be interconnected, of total dimensions 2000 × 540 × 7 mm and 9216 individual resistive pressure sensors 9 × 9 mm each. The operational frequency of the device is within the range of 50 to 100 Hz. The device fulfilled the requirements of i-FAB (international Foot and Ankle Biomechanics community) [54]. The PPAS was calibrated according to the manufacturer’s guidelines before each examination. Before static baropodometric analysis, all participants were informed about the procedure and were asked to adopt a normal, relaxed-standing, bipedal position on the platform; each measurement lasted 20 seconds. The data of baropodometric analysis of each individual were collected using the software “Biomech Studio” Version 1 (Loran Engineering Srl) [53] (Figure 3). The total average plantar pressure (TAPP) was calculated for each foot, denoted as total average affected plantar pressure (TAAPP) and total average unaffected plantar pressure (TAUPP) for the operative and nonoperative side, respectively.

### 2.6. Functional Outcomes

Self-reported functional outcome scores were evaluated through a national validated version of KOOS [55] at the same pre- and postoperative intervals. KOOS is an extension of the WOMAC, originally created and validated in English for patients undergoing TKA [56,57], and has demonstrated its validity, reliability, and responsiveness as a self-administered assessment tool. It has also undergone validation in numerous languages, and it is used extensively in clinical practice and is widely recognized and commonly utilized in knee surgery research [55].

KOOS questionnaire and the baropodometry analysis were performed prior to FTA measurements by the same examiner who did not participate in any other evaluation of the participants. All the data were collected in a Microsoft Excel [58] spreadsheet (Microsoft Office 365, version 2310, Redmond, WA, USA).

### 2.7. Statistical Analysis

The intra-class correlation coefficient (ICC) was calculated to evaluate the agreement of inter- and intra-observer FTA measurements. The Kolmogorov–Smirnov test was used to evaluate the normal distribution of our dataset regarding KA in comparison to KOOS and average PP pre- and postoperatively. The preoperative results showed that the VL dataset significantly deviated from a normal distribution. Postoperatively, the VL group had only two participants showing inconclusive results. The homogeneity assumption was rejected according to the Kolmogorov–Smirnov test. Quantitative variables were expressed as mean values with standard deviation (SD), while categorical and ordinal variables were expressed as absolute and relative frequencies. Fisher’s exact test was used to compare proportions. Paired Student’s *t*-tests and McNemar tests were used for time comparisons. Pearson’s correlation coefficients (r) were used to explore the association between continuous variables, and Spearman’s correlation coefficients (rho) were used for continuous and ordinal variables. One-way analysis of variance (one-way ANOVA) was computed for the comparison of mean values between different groups, and post hoc pairwise comparisons were conducted using the Games–Howell test with a Bonferroni correction. All reported *p* values are two-tailed. Statistical significance was set at *p* < 0.05, and analysis was conducted using IBM SPSS Statistics (version 28.0.1.1(14); IBM Corporation, Armonk, NY, USA) [59].

## 3. Results

There was high intra- and inter-observer agreement on the FTA measurements, with a minimum intra-observer ICC of 0.852 (observer 2, postoperative measurements) and a maximum of 0.890 (observer 1, preoperative measurements). The inter-observer reliability was 0.920 and 0.876 for pre- and postoperative measurements, respectively (Table 2).

### 3.1. KA and KOOS

Before TKA, the KA of the AKJs was determined as VL in 12 cases, N in 5, and VR in 15 based on the FTA measurements. After the TKA, the percentages of KA changed significantly. Only two knees were classified as VL and five as VR, while the vast majority (*n* = 25) (*p* = 0.001) were neutral (Table 3). An insignificant average correction of −0.69° ± 4.12° in the preoperative FTA was measured at the sixth month after TKA. All measured values are shown in Table 4.

We noted significant improvements in the postoperative KOOS values as it was expected (*p* < 0.001) (Table 4). There was a strong positive correlation between pre- and postoperative KOOS scores (r = 0.765; *p* < 0.001). No statistically significant correlations were observed between FTA and KOOS changes (Table 5). To further explore the relationships between the KOOS and FTA, we investigated the correlation between KOOS difference (KOOS df) and absolute FTA difference (*abs* FTA df). The analysis revealed no correlation between these variables (r = −0.07; *p* = 0.702) or correlation of *abs* FTA df with any other variables of interest (Table 5).

There were no significant differences in KOOS scores between KA pre- and postoperatively (*p* > 0.05), (Table 6). Similarly, ANOVA results indicate that there were no significant differences in KOOS scores between different types of KA (VL, N, VR) pre- and postoperatively (*p* > 0.05) (Table 7).

### 3.2. PPs and KA

Significant changes were observed in TAAPP and TAUPP measurements after TKA. TAAPP significantly increased postoperatively (mean change (SD) = 18.60 (47.71); *p* = 0.035), while the opposite trend was observed in TUAPP (mean change (SD) = 51.55 (63.8); *p* < 0.001). Changes were also observed in specific regions of the foot as presented in Supplementary Files. There was a significant increase in the affected limb forefoot (FF) APP following TKA (mean change (SD) = 15.82 (22.28); *p*< 0.001). In contrast, the lateral heel region (LH) exhibited a significant reduction in average PP (mean change (SD) = −9.5 (23.01); *p* = 0.026). No other regions of the affected foot showed statistically significant postoperative changes.

Statistically significant reductions in average PPs were observed on the third, fourth, and fifth metatarsals (M3, M4, and M5) of the unaffected limb compared to the preoperative values (*p* = 0.033, *p* = 0.012, and *p* < 0.01, respectively). Conversely, the FF region significantly decreased in APP postoperatively (*p* < 0.001). In contrast, the midfoot region (MF) APP demonstrated a statistically significant increase following the surgery (*p* < 0.001).

There were no significant differences in TAAPP and TAUPP measurements between KA pre- and postoperatively (*p* < 0.05) (Table 6). ANOVA results showed no significant differences in TAPP and TAUPP pre- and postoperative measurements between different types of KA (*p* > 0.05) (Table 7).

### 3.3. PPs and KOOS

TAAPP and TAUPP preoperatively (pre) correlated weakly positively with the KOOS pre (r = 0.312, *p* = 0.082; r = 0.24, *p* = 0.186). Similarly, postoperative (post) values of TAAPP and TAUPP showed the same trend with KOOS pre (r = 0.228, *p* = 0.208; r = 0.261, *p* = 0.149). TAUPP pre/post displayed weak nonsignificant negative correlations with KOOS df (r = −0.207, *p* = 0.256, and r = −0.174, *p* = 0.341, respectively) (Table 6). Weak and nonsignificant correlations were found between KOOS df, TAAPP, and TUAPP pre- and postoperative differences (TAAPP df, TUAPP df). These findings are consistent with the existing literature, which suggests no significant correlation between coronal alignment and the final clinical outcome, particularly when considering patient-reported outcome measurements [18,20,58,59,60]. In our study, higher preoperative KOOS scores were associated with higher postoperative KOOS scores, suggesting a positive functional outcome after TKA in patients with previously better functional status according to KOOS (Table 6). This suggests, despite the fact that it was not initially hypothesized, that preoperative functional status is a strong predictor of the short-term functional outcome after unilateral TKA in our study population.

## 4. Discussion

According to our analysis, KA did not have a significant impact on the observed pre- and postoperative differences in KOOS, TAAPP, or TAUPP values (Table 6 and Table 7). The correlations between the FTA, KA, and the KOOS pre- and postoperatively were not statistically significant (Table 5), which aligns with the results of studies mainly reporting KOOS outcomes following TKA [61]. The *abs* FTA df did not show significant associations with any variables in our dataset, suggesting that absolute FTA correction may not strongly influence functional outcomes [18,20,60,62,63] or average PP changes (Table 5). Moreover, the corrections in knees with mild deformity do not affect foot loading patterns, which can be explained by the subtalar joint adapting to loading changes [64].

Coronal alignment after TKA can be assessed by the FTA, especially, when only short knee film radiographs are available. Neutral alignment has been defined in three different ranges, according to previous studies; an FTA between 2.4° and 7.2° according to Ritter et al. [50], 3° and 7.5° according to Kim et al. [65], or between 4° and 9° suggested by Morgan et al. [66]. The variations in ranges of neutral alignment might influence the accuracy of the method [67]. However, the difficulty in obtaining full-length films for estimating the hip–knee–ankle angle (HKA) leads to the use of the FTA with acceptable results [51]. Shang et al. [67] found that the postoperative FTA and HKA had a good correlation (r = 0.86) in short knee standing X-rays. Furthermore, Pan et al. [68] reported that for outpatient follow-up, the FTA on non-weight bearing short knee radiographs (NWB SKR) is an acceptable means for classifying knee alignment (varus, neutral, or valgus). While concerns might be raised regarding the use of full-length X-rays, we believe that our methodology aligns with our specific research context.

The most important baropodometric finding in this report was the significant transfer of the PPs to the operated limb by six months after TKA with a reduction in TAUPP on the contralateral side. This is consistent with the majority of published papers in this field [5,11,47,48]. However, there are some conflicting reports, indicating increased loading on the affected foot preoperatively that normalizes after the TKA [34], while others report similar pre- and postoperative loading patterns according to the PP measurements [69,70]. One possible explanation for these differences might be the controversial accuracy of the baropodometry measurements. They can be subjective to some extent, as they can depend on data processing protocols, problems with patients’ behavior, calibration protocols, and differences in the equipment (platforms and/or sensors) [32,71]. These problems hinder the development of a reliable database of baropodometry outcome measurements. The variability of measured values in baropodometric reports (pressure, force, average force/pressure, peak force/pressure) and the lack of a common interpretation of the graphic features make the comparison of results challenging, and it is difficult to draw definitive conclusions from the existing body of research.

By isolating average PPs in specific regions of the foot, baropodometry can provide very interesting results. Our study analysis showed a significant increase in average PP in the FF following TKA, which agrees with the literature [29,34,72], indicating an improvement in weight-bearing ability with simultaneous transfer of the pressures in this region. Our findings suggest that the surgical intervention positively impacted the static load distribution and force patterns on the forefoot. Conversely, the LH region showed a significant average PP reduction, suggesting a weight distribution shift away from this area. This change in HF average PP distribution may be partially explained by the preoperative proportions of VR knees in our study group. VR knees mainly distribute PP to the lateral side of the affected foot as a result of the adaptive valgus deformity of the HF, and it has been shown to have an inverse relationship with the KA after TKA [33].

Baropodometry is considered an objective measurement; however, its results are complex and require specialized knowledge for accurate interpretation. Back in 1997, Rosenbaum and Becker [32] outlined the subjective influences on the method which include patient and technical factors.

Based on the results of our study, performing static baropodometry solely for predicting or evaluating the short-term clinical outcome of unilateral TKA may not be warranted. Static baropodometric examination should not be used as a standalone screening test in KOA patients after unilateral TKA to assess clinical results. Knee surgeons and rehabilitation clinicians should consider a broader range of factors when assessing and predicting subjective clinical outcomes in patients.

This is the first prospective study examining baropodometric analysis, KA, and patient self-reported functional outcome scores after unilateral TKA. Pre- and postoperative KOOS results indicated a statistically significant change, as expected, but this change was not statistically related to the FTA changes and static baropodometric results. Consequently, although differences in alignment may not exhibit a strong association with KOOS outcomes and the findings of static baropodometry post-TKA, this parameter could still hold significance for implant survival [50].

### Limitations

This study has some limitations. The primary limitation lies in the relatively modest sample size of patients under study. However, it is worth highlighting that our study encompasses a larger number of patients when compared to the quantity indicated by the pre-hoc power analysis. Additionally, it is worth acknowledging that potential concerns could arise regarding the utilization of short knee weight-bearing radiographs. Nonetheless, we assert that our chosen methodology is in harmony with the unique research context of our study. Moreover, the discourse surrounding the comparison between full-length and short knee radiographs has been extensively deliberated upon in our discussion section.

Furthermore, all issues that are listed in our discussion section regarding the controversial accuracy of baropodometry are potential limitations, as in all studies on the same subject. On the other hand, the study strengths include the prospective, single-center design, with all procedures performed by the same surgeon using the same implant and with all patients following the same rehabilitation protocol. Moreover, our methodology summarizes both pre- and postoperative measurements of all the parameters under investigation, with a meticulous independent evaluation of the results reducing the risk of bias. Finally, for the baropodometric analysis, we used a multi-platform PPAS that fulfills the requirements of the i-FAB (international Foot and Ankle Biomechanics community) [53], a fact that is not clearly stated in the majority of studies regarding baropodometry.

## 5. Conclusions

In conclusion, our study underscores the intricate interplay between knee alignment, self-reported functional outcomes, and static baropodometric analysis after unilateral TKA. Our findings suggest that while KA, as measured by the FTA, and static baropodometry provide valuable insights, they may not be definitive predictors of short-term clinical outcomes reported by patients. Other factors such as preoperative functional status and patients’ perception of their knee condition appear to wield stronger influence. While static baropodometry remains an objective measurement tool, our findings caution against relying solely on its results for short-term clinical outcome predictions following unilateral TKA. The lack of significant correlation between pedobarometric results and other variables highlights the need for investigations to identify other factors influencing static APP changes and to understand their implications for functional outcomes and patient rehabilitation. By recognizing the limitations and complexities of baropodometry, clinicians can better tailor post-TKA rehabilitation strategies and patient management to achieve more accurate and comprehensive patient care.

## Figures and Tables

**Table 1:** Sample characteristics.

	N (%)
Sex	
Females	22 (68.8)
Males	10 (31.3)
Age (years), mean (SD)	72.8 (6.6)
BMI (kg/m^2^), mean (SD)	27.1 (2.0)
BMI	
Normal	4 (12.5)
Overweight	26 (81.3)
Obese	2 (6.3)
Affected knee joint	
Left	17 (53.1)
Right	15 (46.9)
KLC	
3	9 (28.1)
4	23 (71.9)

BMI: Body Mass Index; KLC: Kellgren–Lawrence Classification.

**Table 2:** Intra-class coefficients (ICCs) for agreement in FTA measurements.

Intra-Observer	Pre	95% CI	*p*	Post	95% CI	*p*
*Observer 1*	0.890	0.788–0.945	<0.001	0.866	0.743–0.932	<0.001
*Observer 2*	0.881	0.770–0.940	<0.001	0.852	0.718–0.925	<0.001
Inter-observer	0.920	0.842–0.960	<0.001	0.876	0.762–0.938	<0.001

ICC: intra-class coefficient; 95% CI: 95% confidence Interval; pre: preoperative; post: postoperative.

**Table 3:** Changes in KOOS, FTA, and knee alignment groups.

	Pre	Post	*p*
	Mean (SD)	Mean (SD)	
KOOS	34.40 (7.98)	60.93 (6.9)	<0.001 +
FTA	176.26 (5.37)	175.57 (1.73)	0.305 +
KA, N (%)			
VL	12 (37.5)	2 (6.3)	0.001 ++
N	5 (15.6)	25 (78.1)	
VR	15 (4.9)	5 (15.6)	

+ Paired *t*-test; ++ McNemar test; *p* significant at the 0.05 level; KOOS: Knee Injury and Osteoarthritis Outcome Score, FTA: Femorotibial Angle, KA: Knee Alignment, VL: Valgus, N: Neutral, VR: Varus. FTA values in degrees (°).

**Table 4:** FTA, KOOS, TAAPP, and TAUPP measurements pre- and postoperatively.

	Mean	Min	Max	Std.	Range
FTA pre	176.26	169.43	187.45	5.37	18.02
FTA post	175.57	172.38	180.10	1.74	7.72
FTA df	−0.69	−9.77	4	4.12	14.23
*abs* FTA df	3.68	1.13	9.78	1.86	8.65
KOOS pre	33.94	20	46	8.21	26
KOOS post	60.59	46	74	7.12	28
KOOS df	26.66	16	37	5.19	21
TAAPP pre	420.13	128	729	111.11	601
TAAPP post	480.28	167	713	127.87	546
TAAPP df	60.16	−16	197	53.22	213
TAUPP pre	505.47	332	715	110.56	383
TAUPP post	454.19	298	675	96.57	377
TAUPP df	−51.28	−183	72	59.81	255

df: Difference (post(value)–pre(value)), *abs*: Absolute, TAAPP: Total Average Affected Plantar Pressure, TAUPP: Total Average Unaffected Plantar Pressure. FTA values in degrees (°). Plantar pressure measurements in kilopascal (kPa).

**Table 5:** Pearson’s correlation coefficient (r) of quantitative measurements of interest.

		FTA Pre	FTA Post	FTA df	*abs* FTA df	KOOS Prs	KOOS Post	KOOS df	TAAPP Pre	TAAPP Post	TAUPP Prs	TAUPP Post	TAUPP df
**KOOS pre**	(r)	0.107	0.026	−0.129	0.179	--							
	*p*	0.56	0.886	0.483	0.327								
**KOOS post**	(r)	0.000	−0.174	−0.073	0.154	0.765 **	--						
	*p*	0.999	0.341	0.691	0.4	<0.001							
**KOOS df**	(r)	−0.165	−0.271	0.1	−0.07	−0.519 **	0.153	--					
	*p*	0.368	0.133	0.585	0.702	0.002	0.402						
**TAAPP pre**	(r)	0.304	0.325	−0.259	0.327	0.312	0.259	−0.135	--				
	*p*	0.091	0.069	0.152	0.068	0.082	0.153	0.461					
**TAAPP post**	(r)	0.312	0.341	−0.264	0.285	0.228	0.165	−0.131	0.894 **	--			
	*p*	0.082	0.056	0.145	0.113	0.208	0.366	0.475	<0.001				
**TAUPP pre**	(r)	0.313	0.461 **	−0.213	0.159	0.24	0.122	−0.207	0.791 **	0.840 **	--		
	*p*	0.081	0.008	0.241	0.385	0.186	0.507	0.256	<0.001	<0.001			
**TAUPP post**	(r)	0.319	0.420 *	−0.238	0.252	0.261	0.171	−0.174	0.786 **	0.722 **	0.830 **	--	
	*p*	0.076	0.017	0.189	0.165	0.149	0.35	0.341	<0.001	<0.001	<0.001		
**TAAPP df**	(r)	0.005	0.068	0.022	−0.091	−0.034	−0.087	−0.063	0.269	0.308	0.225	0.258	--
	*p*	0.977	0.712	0.906	0.621	0.852	0.635	0.731	0.137	0.087	0.215	0.154	
**TAUPP df**	(r)	0.056	0.161	−0.006	−0.112	0.018	−0.052	−0.096	0.173	0.363 *	0.478 **	−0.093	−0.004
	*p*	0.759	0.378	0.976	0.541	0.924	0.779	0.603	0.342	0.041	0.006	0.611	0.985

** Correlation is significant at the 0.01 level (2-tailed). * Correlation is significant at the 0.05 level (2-tailed).

**Table 6:** Spearman’s correlation coefficient (rho) between continuous and ordinal variables of interest.

KA	KOOS Pre	KOOS Post	TAAPP Pre	TAPP Post	TAUPP Pre	TAUPP Post
pre (rho)	0.138	0.012	0.217	0.209	0.259	0.128
*p*	0.450	0.950	0.233	0.251	0.153	0.485
post (rho)	−0.112	−0.145	0.253	0.212	0.337	0.182
*p*	0.540	0.428	0.162	0.244	0.059	0.320

Correlation is significant at the 0.01 level (2-tailed).

**Table 7:** KOOS, TAAPP, and TAUPP pre- and postoperative values by knee alignment.

			KOOS			TAAPP			TAUPP	
	KA (*n*)	Mean	SD	*p* ANOVA	Mean	SD	*p* ANOVA	Mean	SD	*p* ANOVA
pre	VL (13)	33.25	7.73	0.817	415.73	81.86	0.1	472.20	114.72	0.579
	N (4)	34.6	7.47		386.29	69.30		516.56	154.23	
	VR (15)	35.26	8.73		477.5	104.87		515.37	95.86	
post	VL (2)	58	5.65	0.206	408.95	86.33	0.43	426.00	151.32	0.189
	N (25)	62.08	6.31		478.49	123.65		455.58	98.26	
	VR (5)	56.40	9.18		533.38	85.20		541.86	86.23	

*p* ANOVA significant at the 0.05 level.

## Data Availability

The dataset presented and analyzed in this study has been uploaded to FigShare.com (accessed on 28 June 2023). DOI will be available upon request to the corresponding author.

## Supplementary Materials

The following supporting information can be downloaded at https://www.mdpi.com/article/10.3390/jcm12216917/s1, Table S1: Regional average plantar pressure measurements in the affected and unaffected limb before and six months after TKA.
