# Supplementary material for: Static Baropodometry for Assessing Short-Term Functional Outcome after Unilateral Total Knee Arthroplasty: Exploring Correlation between Static Plantar Pressure Measurements and Self-Reported Outcomes
Source: J Clin Med. 2023 Nov 3;12(21):6917. doi: 10.3390/jcm12216917 (PMC10650917; doi:10.3390/jcm12216917)
Supplement: Supplementary file 1 [file jcm-12-06917-s001.zip › jcm-2511411-supplementary.pdf]

Table S1. Regional Average plantar pressure measurements in the affected an unaffected limb before and six months after TKA.

	Affected				Unaffected			
	**Post**	**Pre**	**Mean (SD)**	**p**	**Post**	**Pre**	**Mean (SD)**	**p**
T1	11.70	10.41	1.29 (11.37)	0.524	13.8	15.87	-2.07 (8.98)	0.202
T2-5	13.55	10.75	2.80 (11.36)	0.173	15.82	16.59	-0.77 (8.86)	0.625
M1	47.84	44.18	3.66 (33.99)	0.547	41.49	44.71	-3.22 (14.02)	0.203
M2	47.63	43.84	3.78 (24.14)	0.382	47.97	50.25	-2.28 (12.38)	0.305
M3	50.13	40.69	9.44 (28.70)	0.072	49.4	55.37	-5.97 (15.14)	0.033*
M4	47.44	48.16	-0.72 (16.42)	0.806	52.34	58.84	-6.50 (13.69)	0.012*
M5	44.69	49.13	-444 (28.70)	0.388	49.90	57.22	-7.31 (10.38)	<.001*
MF	47.88	43.03	4.84 (26.60)	0.311	59.16	44.21	14.94 (19.74)	<.001*
MH	85.59	78.16	7.44 (23.66)	0.085	80.12	76.53	3.60 (23.13)	0.386
LH	70.56	80.06	-9.50 (23.01)	0.026*	76.68	81.57	-4.88 (23.91)	0.257
FF	262.97	247.15	15.82 (22.28)	<0.001*	270.75	298.87	-28.13 (41.96)	<.001*
HF	156.16	158.22	2.06 (25.98)	0.657	161.69	153.21	8.48 (26.37)	0.079
APP	467.00	448.40	18.60 (47.71)	0.035*	468.19	519.73	-51.55 (63.80)	<.001*

* Paired Samples Test significant at the 0.05 level (2-tailed). T1: Big toe, T2-5: second to fifth toes, M(1-5): Metatarsal, MF: Midfoot, MH: Medial heel, LH: Lateral heel, HF: Hindfoot, APP: Average plantar pressure, Measurements of plantar pressure in kilopascal (kPa)
